# Comment on “Accuracy and usability of a diagnostic decision support system in the diagnosis of three representative rheumatic diseases: a randomized controlled trial among medical students”

**DOI:** 10.1186/s13075-022-02770-5

**Published:** 2022-04-04

**Authors:** Stephen Gilbert, Paul Wicks

**Affiliations:** 1 Ada Health GmbH, Karl-Liebknecht-Str. 1, 10178 Berlin, Germany; 2 The Else Kröner Fresenius Center for Digital Health, University Hospital Carl Gustav Carus Dresden, Technische Universität Dresden, Dresden, Germany

Dear Editor,

We have several comments on the recent publication of [2]. Knitza et al. [2] tested Ada’s consumer facing app, which was carefully designed for the intended purpose of providing layperson users the opportunity to find out what might be causing their health issues. Over many years, it has been optimized for the layperson using the principles of user-centered design, a process focused on user needs, user characteristics, and end-user testing of the user–machine interface [1]. Ada’s consumer app is definitively not a ‘diagnostic decision support system’ (DDSS) and was not designed to provide usability to the health care professionals (HCPs) as Knitza et al. sought to investigate. A foundational principle of medical device usability and regulatory science is that use errors occur as a result of inappropriate design for the *intended user* or for the *intended purpose* and that these lead to safety issues, reduced performance and reduced clinical outcomes [1]. Every aspect of Ada’s consumer app, including its interface and its means of gathering symptoms, is fundamentally different from an HCP-facing DDSS. Ada’s consumer app asks a series of questions in easily accessible language, avoiding specialist terminology. The lay user cannot provide professional-level medical information that requires a healthcare professional to discern. It does not allow the user to direct or short-cut any aspect of the question flow, as they are not qualified to do so. By contrast, a DDSS for highly trained professionals in modern healthcare settings would take a different approach.

Specifically, Ada has followed user-centered design principles to develop and test an under development/prototype HCP-facing DDSS system that was not utilized in Knitza et al.’s research. This uses the same underlying Ada medical intelligence platform but utilizes a more appropriate interface. It also allows HCP-level clinical symptom and test-finding information to be entered. We have conducted careful formative usability research in collaboration with academic expert centers, and this has contributed to the iterative user-centered design process [4]. The HCP-facing DDSS has shown potential for assisting early rare disease diagnosis [3], with the potential for economic benefits to health care systems [5].

The usability and accuracy of a medical device are user and use-case determined, so unfortunately, no useful interpretation of the results in Knitza et al.’s [2] research can be made.

## Data Availability

Not applicable

## Abbreviations

DDSS: Diagnostic decision support system
HCP: Health care professional
